# RETRACTION: Treatment of Diabetic Foot With Moxibustion: Clinical Evidence From Meta‐Analysis

**DOI:** 10.1111/iwj.70655

**Published:** 2025-04-23

## Abstract

**RETRACTION:** JiY. , ZhangY. , WuR. , WangT. , WangJ. , LiuZ. , and LiuW. , “Treatment of Diabetic Foot With Moxibustion: Clinical Evidence From Meta‐Analysis,” International Wound Journal 21, no. 2 (2024): e14791, 10.1111/iwj.14791.38361252 PMC10869878

The above article, published online on 15 February 2024, in Wiley Online Library (http://onlinelibrary.wiley.com/), has been retracted by agreement between the journal Editor in Chief, Professor Keith Harding; and John Wiley & Sons Ltd. Following an investigation by the publisher, all parties have concluded that this article was accepted solely on the basis of a compromised peer review process. The editors have therefore decided to retract the article. The authors did not respond to our notice regarding the retraction.

**RETRACTION:** Y. Ji , Y. Zhang , R. Wu , T. Wang , J. Wang , Z. Liu , and W. Liu , “Treatment of Diabetic Foot With Moxibustion: Clinical Evidence From Meta‐Analysis,” International Wound Journal 21, no. 2 (2024): e14791, 10.1111/iwj.14791.38361252 PMC10869878

The above article, published online on 15 February 2024, in Wiley Online Library (http://onlinelibrary.wiley.com/), has been retracted by agreement between the journal Editor in Chief, Professor Keith Harding; and John Wiley & Sons Ltd. Following an investigation by the publisher, all parties have concluded that this article was accepted solely on the basis of a compromised peer review process. The editors have therefore decided to retract the article. The authors did not respond to our notice regarding the retraction.

